# Effectiveness of a video-based intervention for COVID-19 vaccine acceptance among individuals with mental disorders: a randomized online experiment

**DOI:** 10.3389/fpubh.2026.1821349

**Published:** 2026-06-23

**Authors:** Daniel Huth, Severin Hennemann, Michael Witthöft, Anne-Kathrin Bräscher

**Affiliations:** Department of Clinical Psychology, Psychotherapy, and Experimental Psychopathology, Johannes Gutenberg University Mainz, Mainz, Germany

**Keywords:** COVID-19 pandemic, educational intervention, health beliefs, psychopathology, risk perceptions, vaccination willingness

## Abstract

**Introduction:**

During the COVID-19 pandemic, vaccine hesitancy and low risk awareness were also observed in high-risk groups, such as individuals with mental disorders. This study evaluated an educational video aimed at increasing COVID-19 vaccine acceptance in this population.

**Methods:**

A randomized online experiment with 639 participants from the German general population (*M*_Age_ = 37.04; 77.8% female; 24.4% self-reporting a mental disorder) was conducted during the second year of vaccine distribution. Participants were randomly assigned to either the educational video or a control group (no video). Outcomes included vaccination willingness, threat perceptions, and vaccine-specific beliefs. Moderation analyses examined differential effects for diagnostic status and internalizing psychopathology.

**Results:**

The intervention significantly increased awareness of the importance of vaccination for individuals with mental disorders (*p* = 0.033, *d* = 0.17), perceived severity of COVID-19 (*p* = 0.010, *d* = 0.21), and deliberative decision-making (*p* = 0.015, *d* = 0.19). However, it did not significantly increase vaccination willingness or change vaccine-specific beliefs. Moderation analyses showed that participants with a self-reported mental disorder had greater perceived susceptibility (*p* = 0.032, *d* = 0.36), but internalizing psychopathology did not influence outcomes.

**Discussion:**

While the video improved awareness and threat perceptions, further efforts are needed to address vaccine hesitancy and motivate behavior change in high-risk populations.

## Introduction

The effectiveness of vaccination in preventing severe disease outcomes of the acute coronavirus disease 2019 (COVID-19) is well-documented, with substantial evidence suggesting a reduction in both morbidity and mortality among vaccinated individuals (1, 2). The contribution of booster vaccinations in sustaining and augmenting immune protection has been further corroborated, emphasizing their critical role in maintaining long-term immunity (3–5). Despite these benefits, vaccine coverage fell short of the levels required to achieve herd immunity in many countries (4, 6, 7). As the pandemic advanced, vaccination rates had plateaued, with booster uptake notably lagging behind rates of basic immunization. Thus, the need for public health interventions to address vaccine hesitancy effectively remained relevant throughout the pandemic, particularly for populations at high risk.

Individuals with mental disorders, such as depression, anxiety, or substance-related disorders, represent such a high-risk group. Extensive evidence from electronic health record data has established a robust association between mental disorders across all diagnostic categories and an increased risk of SARS-CoV-2 infection, severe COVID-19 outcomes, and increased mortality (8, 9). With an estimated 12-month prevalence of any mental disorder in 25%−30% of the German general population (10), this group constitutes a substantial proportion of those requiring targeted protective measures. As a result, prioritizing individuals with mental disorders for COVID-19 vaccination has been advocated and, in some countries, implemented during vaccine distribution (11–13).

Evidence on COVID-19 vaccine acceptance among individuals with mental disorders is, however, mixed. A scoping review has highlighted lower vaccination rates and increased vaccine hesitancy among individuals with mental disorders (14). For instance, a comparison between psychiatric patients and the general population in Denmark found that a mental health diagnosis was significantly associated with a 4.7% lower willingness to receive the COVID-19 vaccine following the initiation of vaccine distribution (15). Conversely, a recent longitudinal study investigating COVID-19 vaccine uptake during the initial 2 years of vaccine distribution in Germany found no evidence of reduced vaccination rates among individuals with mental disorders. However, the study neither found evidence of accelerated uptake nor more favorable psychological determinants within this population, which would have been desirable given their increased vulnerability to COVID-19. Notably, individuals with mental disorders exhibited a lower perceived risk of infection, suggesting a lack of awareness regarding their heightened susceptibility (16).

Several theoretical frameworks exist aiming to explain the adoption of preventive health behaviors [e.g., (17, 18)]. These frameworks consistently emphasize the role of psychological antecedents, defined as the thoughts and feelings that precede and influence the health behavior in question (19). Across various models, these antecedents typically include perceptions of the preventable disease (e.g., perceived severity or susceptibility), evaluations of the preventive measure (e.g., confidence in the safety and effectiveness of the measure), and relevant social processes. A framework integrating these dimensions in the context of vaccination is the 5C model (20). Beyond confidence, the model further considers the belief that vaccination is unnecessary due to limited threat of the preventable disease (i.e., complacency), perceived barriers to vaccination (i.e., constraints), cost-benefit evaluations (i.e., calculation), and perceptions of collective responsibility to contribute to an individual's likelihood of getting vaccinated. These determinants have been empirically linked to COVID-19 vaccination and booster uptake indicating that these factors represent suitable targets for interventions aimed at promoting vaccination (21–23).

Existing measures to promote vaccination can be classified as indirect, targeting perceptions of disease or vaccination, or direct, providing incentives or improving access independent of psychological determinants (23). Pre-pandemic evidence suggests that indirect measures, such as those designed to enhance risk appraisals, yield small-to-moderate effects on vaccine acceptance across different vaccines (24). In contrast, direct interventions generally demonstrate greater efficacy but are frequently constrained by feasibility and implementation challenges (25–27). Efforts to increase COVID-19 vaccine uptake have predominantly relied on communication and educational strategies, often disseminated via digital platforms (28). Recommendations for effective communication emphasize addressing common health beliefs, ensuring transparency, and acknowledging vaccine-related concerns (29). Overall, meta-analytic findings indicate that behavioral interventions exert a small but statistically significant effect on COVID-19 vaccine uptake. Importantly, their effectiveness does not appear to be moderated by the timing of implementation (30).

As the pandemic progressed, recommendations to address stagnating vaccination rates increasingly emphasized the need for tailored communication strategies, with a particular focus on vulnerable groups (6). Therefore, in this randomized online experiment we examined the effectiveness of a video-based educational intervention designed to promote the willingness to vaccinate against COVID-19. In addition to providing general information addressing common questions and concerns about COVID-19 vaccination, the intervention was specifically tailored to encourage vaccination among individuals with mental disorders. Drawing from evidence of similar interventions (31, 32), we hypothesized that participants in the intervention group (i.e., educational video) report higher vaccine willingness and increased awareness of the heightened necessity of COVID-19 vaccination for individuals with mental disorders than in the control group (i.e., no video). Further, we examined intervention effects on psychological antecedents of vaccination willingness, including disease-related risk perceptions and evaluations of COVID-19 vaccination as conceptualized by the 5C model (20). To evaluate the differential effectiveness within the targeted group, we investigated whether diagnostic status or clinically relevant levels of internalizing psychopathology moderate the effectiveness of the intervention.

## Material and methods

All data, analysis code, and research materials including the video intervention and a translated transcript have been made publicly at PsychArchives and can be accessed at https://doi.org/10.23668/psycharchives.22187. Data were analyzed using R version 4.4.2 (33). This study was not preregistered.

### Participants

A non-probability convenience sample of the German general population was recruited through social media (e.g., Facebook, Twitter), university mailing lists, an information service organization for local support groups and online press releases between April 7, 2022 and April 30, 2022. Despite the tailored design of the intervention, convenience sampling was employed in accordance with a universal preventive strategy, i.e., aiming to reach individuals irrespective of their COVID-19 vaccination or diagnostic status. This sampling approach was selected to approximate conditions of minimal participant screening, thereby reflecting realistic conditions commonly encountered in public health communication efforts where audience control is constrained by limited resources. During the study period, daily new SARS-CoV-2 infections ranged from 26,993 to 188,947 cases in Germany. The Containment and Health Index [CHI; (34)] remained consistently at 41.5, while COVID-19 vaccine coverage, based on first-dose uptake, plateaued at 78%.

Inclusion criteria were at least 16 years of age and the provision of informed consent. As compensation, participants were offered entry into a drawing for five online shopping vouchers worth 20€ each after study completion. Alternatively, psychology students of the University of Mainz were given the opportunity to earn research participation credits.

### Procedure

A randomized controlled online experiment was conducted using SoSci Survey (35). Eligible participants first completed pretest questionnaires assessing sociodemographic and clinical characteristics, history of SARS-CoV-2 infection, and COVID-19 vaccination status. Subsequently, participants were randomly assigned to either the intervention (educational video) or control condition (no video) through simple 1:1 randomization implemented within the survey platform (i.e., random draw with discarding). No video was provided to the control condition, as the development of neutral video stimuli poses methodological challenges, and the absence of such manipulation reflects the naturalistic state of control participants.

In the intervention condition, participants received a 5:41-min video-based educational intervention. The content was developed based on established health behavior frameworks (17, 18, 20) and COVID-19 vaccination communication guidelines (29). The video emphasized the risks associated with COVID-19 and addressed common concerns regarding the safety and effectiveness of COVID-19 vaccines. It also discussed societal aspects, including collective responsibility and social norms, highlighted the benefits of vaccination, and provided cues to action. Additionally, the intervention emphasized the importance of COVID-19 vaccination for individuals with mental disorders by referencing empirical findings of higher susceptibility to infection and more severe disease outcomes in this population, and by describing psychoneuroimmunological mechanisms. A licensed clinical psychologist presented all information to enhance credibility.

Following the video, participants in the intervention condition completed post-intervention questionnaires to assess vaccine-related primary and secondary outcomes. Participants in the control condition completed post-intervention questionnaires immediately after the pretest and were then presented with the video.

The study protocol was reviewed and approved by the ethics board of the Department of Psychology at the University of Mainz (2022-JGU-psychEK-S015, April 7, 2022). All participants provided informed consent via the survey platform after study procedures have been fully explained and prior to the initiation of any study procedures.

### Measures

#### Primary outcomes

Vaccination willingness was assessed by a single item (“If you were offered the COVID-19-vaccination next week, how would you decide?”) on a seven-point Likert scale from 1 (“definitely don't vaccinate”) to 7 (“definitely vaccinate”). If no further vaccination was indicated at the time of survey participation, participants were engaged to answer the item in case that another vaccine dose was officially recommended for them next week.

Awareness of the importance of COVID-19 vaccination for people with mental disorders was assessed by a single item (“How important do you think vaccination against COVID-19 is for people with a mental disorder?”) on a seven-point Likert scale from 1 (“not important at all”) to 7 (“very important”).

#### Secondary outcomes

##### Disease-related threat perceptions

Perceived severity of and perceived susceptibility to COVID-19 were assessed each with a single item (i.e., “An infection with the coronavirus is dangerous.”, “I consider myself susceptible to a coronavirus infection.”) on a seven-point Likert scale from 1 (“do not agree at all”) to 7 (“totally agree”).

##### 5C scale

The short version of the 5C scale was administered to assess vaccine-specific beliefs according to the 5C model of vaccination readiness [i.e., confidence, complacency, constraints, calculation, collective responsibility; (20)]. The short version comprises five statements covering the five dimensions (one item per dimension) that are rated on a 7-point scale (1 = strongly disagree, 7 = strongly agree). As in previous studies [e.g., (27)], we adapted the items to the context of COVID-19 vaccines. Because dimensions display rather distinct constructs, analyses are conducted at the item-level.

#### Psychopathology and related information

##### Mental disorder diagnostic status

Mental disorders were assessed via self-report by asking participants “Are you currently experiencing a mental illness?”. Participants could select from a list of common mental disorder categories (e.g., major depression, bipolar disorder, anxiety disorder, post-traumatic stress disorder, obsessive-compulsive disorder, psychotic disorders, substance use disorder, somatic distress disorder, eating disorder, personality disorder) using a dichotomous response format (0 = not present, 1 = present). For diagnoses not covered by the pre-selected options, a free response format was available. Additionally, participants who reported a mental disorder were asked to specify the source of their diagnosis. In this study, the presence of any self-reported mental disorder diagnosed by a professional (0 = no, 1 = yes) was included in the analyses.

##### Depression and anxiety

Depressive and anxiety symptoms were measured using the Patient Health Questionnaire-4 [PHQ-4; (36)], which includes two core symptoms each for depression (i.e., “having little interest or pleasure in doing things” and “feeling down, depressed, or hopeless”) and anxiety (i.e., “feeling nervous, anxious, or on edge” and “not being able to stop or control worrying”). These symptoms were rated for the past 2 weeks on a 4-point scale (0 = “not at all” to 3 = “nearly every day”). For statistical analyses, the PHQ-4 subscales were used to separately measure depressive [PHQ-2; (37)] and anxiety symptoms [GAD-2; (38)]. Scores range between 0 and 6 points, with a proposed cut-off for screening for depression or anxiety of ≥3. The internal consistencies (Spearman-Brown corrected correlations) were ρ = 0.85 and ρ = 0.79 for the PHQ-2 and GAD-2, respectively.

#### Additional measures

Sociodemographic data, including age, gender, and education level were collected by self-report. Participants were also asked about the number of COVID-19 vaccine doses they had received to date and whether they had previously been infected with SARS-CoV-2. We utilized the duration of time spent on the page displaying the video as a proxy measure for intervention exposure.

### Statistical analyses

All tests were two-sided with a significance level of 0.05. Analyses of covariance (ANCOVA) were performed to examine the effect of the video intervention on primary and secondary outcomes. Effect sizes (Cohen's d) for between-group differences were calculated using the pooled standard deviation (SD) of the intervention and control conditions. A positive d value indicates higher outcome values in the intervention condition. According to Cohen (39), *d* = 0.20 is considered a small effect, *d* = 0.50 a medium effect, and *d* = 0.80 a large effect. Moderation analyses were conducted to examine interaction effects of condition with self-reported mental disorders and positive screenings for depression (PHQ-2) and anxiety (GAD-2), i.e., score below 3 vs. score of 3 or higher. Each moderator was analyzed using a separate model containing condition, the respective binary moderator, and the condition × moderator interaction as predictors. Significant interaction effects were further analyzed by estimating the effect of condition for each value of the moderator separately. Age and gender were included as covariates in all models, with age centered at the mean. Additionally, subgroup analyses were performed within a subsample of unvaccinated individuals, applying the same analysis steps described above.

## Results

### Sample characteristics

A total of *n* = 715 (*n*_intervention_ = 336, *n*_control_ = 379) participated in the current study (see Supplementary Figure 1). Prior to data analysis, we removed *n* = 76 (22.6%) participants from the intervention condition due to insufficient intervention exposure (i.e., view time < 80% of video duration; *n* = 61), implausibly high intervention exposure (i.e., view time 3 SDs above mean; *n* = 3), and reports of video malfunction (*n* = 20). Excluded participants did not differ significantly from included participants of the intervention condition regarding sociodemographic and clinical characteristics (see Supplementary Table 1). In total, *n* = 639 (*n*_intervention_ = 260, *n*_control_ = 379) participants remained to be analyzed in the current study. Assuming a significance level of 0.05, the observed sample size of 639 enabled the detection of a small effect with a statistical power of 71.4% in the ANCOVAs including two covariates.

The mean age was 37.04 years (*SD* = 14.85, range 16–81), and 77.8% (*n* = 497), 21.3% (*n* = 136), and 0.9% (*n* = 6) reported being female, male, and non-binary, respectively. Most of the participants were highly educated and indicated a university degree (47.7%, *n* = 305) or university entrance qualification (39.0%, *n* = 249). Among respondents, 92.0% (*n* = 588) reported having received at least one COVID-19 vaccination, with the majority (82.8%, *n* = 529) having been vaccinated three or more times. Further, 24.4% (*n* = 156) reported to have been currently diagnosed with a mental disorder by a professional (for frequencies of diagnostic categories see Supplementary Table 2). We collected no data on race/ethnicity. Table 1 contains detailed information on sociodemographic and clinical characteristics of both study groups. Sociodemographic and clinical characteristics were similar in both groups (*p* > 0.05). Bivariate associations between study variables are depicted in Supplementary Table 3.

**Table 1:** Sociodemographic and clinical characteristics between conditions.

Variables	Video (*n* = 260)	Control (*n* = 379)	Test statistics
Age, years, *M* (*SD*)	36.63 (14.35)	37.33 (15.19)	*t*_(637)_ = 0.59, *p* = 0.557
Gender, *n* (%)			
Female	203 (78.1)	294 (77.6)	χ^2^ = 1.85, *p* = 0.399^a^
Male	53 (20.4)	83 (21.9)	
Other	4 (1.5)	2 (0.5)	
Education, *n* (%)			
Secondary school degree or lower	29 (11.2)	56 (14.8)	χ^2^(2) = 5.30, *p* = 0.071
Higher education entrance qualification	93 (35.8)	156 (41.2)	
University degree	138 (53.1)	167 (44.1)	
Prior SARS-CoV-2 infection, yes, *n* (%)	90 (34.6)	130 (34.3)	χ^2^(1) < 0.01, *p* = 1.000
COVID-19 vaccine doses, *n* (%)			
0	22 (8.5)	29 (7.7)	χ^2^ = 2.58, *p* = 0.643^a^
1	2 (0.8)	5 (1.3)	
2	24 (9.2)	28 (7.4)	
3	197 (75.8)	302 (79.7)	
4	15 (5.8)	15 (4.0)	
Self-reported mental disorder, yes, *n* (%)	66 (25.4)	90 (23.7)	χ^2^(1) = 0.140, *p* = 0.704
Depressive symptoms			
PHQ-2, sum, *M* (*SD*)	1.79 (1.63)	1.80 (1.61)	*t*_(637)_ = 0.12, *p* = 0.901
PHQ-2, clinical^b^, *n* (%)	70 (26.9)	97 (25.6)	χ^2^(1) = 0.08, *p* = 0.776
Anxiety symptoms			
GAD-2, sum, *M* (*SD*)	1.69 (1.54)	1.87 (1.67)	*t*_(637)_ = 1.33, *p* = 0.184
GAD-2, clinical^b^, *n* (%)	59 (22.7)	110 (29.0)	χ^2^(1) = 2.86, *p* = 0.091

^a^Chi-squared test with simulated *p*-values based on 2,000 replicates were computed. ^b^Clinical values are reached at sum ≥3.

### Primary outcomes

Contrary to our hypothesis, no significant effect of the video intervention was observed for vaccination willingness, *F*_(1, 634)_ = 0.28, *p* = 0.598. However, analysis revealed a significant effect of condition on awareness favoring the video intervention, *F*_(1, 634)_ = 4.58, *p* = 0.033, with a near-to-small between-group effect size, *d* = 0.17.

### Secondary outcomes

Analyses of secondary outcomes revealed significant small effects of the video intervention on perceived severity, *F*_(1, 634)_ = 6.70, *p* = 0.010, *d* = 0.21, and calculation (i.e., cost-benefit evaluations), *F*_(1, 634)_ = 5.94, *p* = 0.015, *d* = 0.19, indicating higher values in the intervention condition. No significant differences between conditions were found for the remaining outcomes (*p* > 0.05). Detailed results for group comparisons regarding primary and secondary outcomes can be found in Table 2.

**Table 2:** Observed means and standard deviations for primary and secondary outcomes per condition and between-group comparisons.

Outcomes	Video (*n* = 260)	Control (*n* = 379)	Effect size *d* [95% *CI*]
Primary outcomes			
Vaccination willingness	5.62 (2.09)	5.70 (2.01)	−0.04 [−0.20, 0.12]
Awareness	6.02 (1.59)	5.72 (1.74)	0.17 [0.01, 0.33]^*^
Secondary outcomes			
*Threat perceptions*			
Severity	5.15 (1.51)	4.84 (1.50)	0.21 [0.05, 0.36]^**^
Susceptibility	3.54 (1.82)	3.51 (1.66)	0.02 [−0.14, 0.18]
*5C dimensions*			
Confidence	5.64 (1.70)	5.45 (1.75)	0.10 [−0.06, 0.26]
Complacency	1.69 (1.24)	1.83 (1.44)	−0.10 [−0.26, 0.06]
Constraints	1.18 (0.64)	1.19 (0.64)	−0.01 [−0.17, 0.15]
Calculation	5.62 (1.65)	5.30 (1.79)	0.19 [0.04, 0.35]^*^
Collective responsibility	6.41 (1.14)	6.50 (1.10)	−0.08 [−0.24, 0.07]

All outcomes were measured on a seven-point Likert scale (1–7). Main effects of condition were estimated controlling for age and gender. ^*^p < 0.05, ^**^p < 0.01.

### Moderation analyses

**Figure 1:**
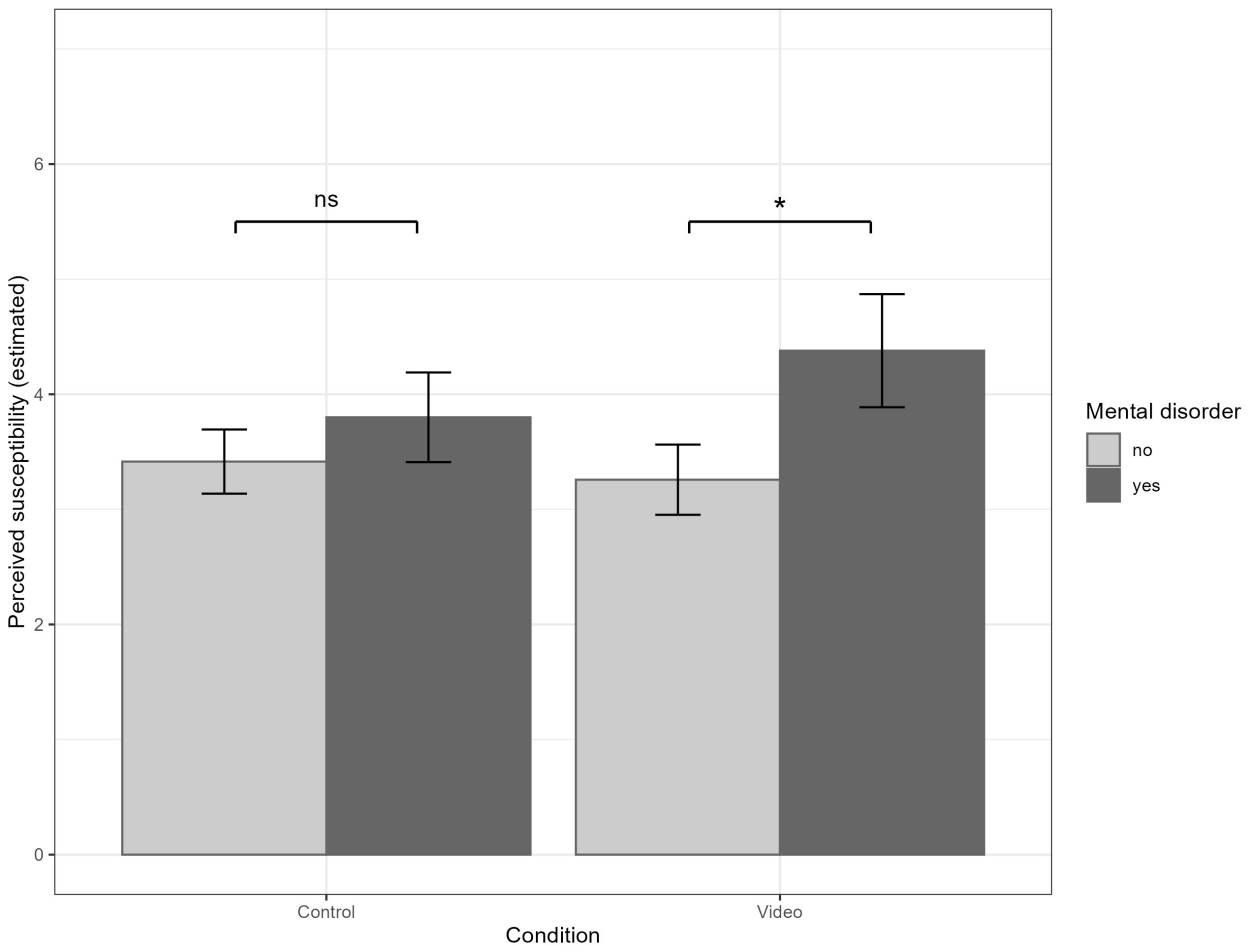
Visualizations of the results testing self-reported mental disorder as a moderator of the outcome perceived susceptibility between intervention and control condition. Estimated marginal means of perceived susceptibility to COVID-19 by condition (video vs. control) and self-reported mental disorder (yes vs. no). Estimates are derived from an analysis of covariance (ANCOVA) including condition, self-reported mental disorder, and their interaction as predictors, controlling for age and gender. Error bars represent 95% confidence intervals. *Post-hoc* tests revealed a significant positive effect of the video intervention on perceived susceptibility only within individuals with a self-reported mental disorder. *p < 0.05, ns, not significant.

Self-reported presence of a mental disorder significantly moderated the effect of condition on perceived susceptibility [interaction effect condition × diagnostic status: β = 0.09, 95% CI (0.02, 0.17), *p* = 0.016; see Figure 1]. *Post-hoc* analyses revealed that the video intervention significantly increased perceived susceptibility for individuals who reported a mental disorder, β = 0.18, 95% CI [0.02, 0.34], *p* = 0.032, *d* = 0.36, but not for individuals without a mental disorder diagnosis, β = −0.05, 95% CI [−0.14, 0.04], *p* = 0.309. No further significant interaction effects of condition with diagnostic status or above-threshold depressive and anxiety symptoms were observed for the remaining outcomes (*p* >0.05; see Table 3).

**Table 3:** Results of the moderation analyses investigating “condition x moderator” effects on COVID-19 vaccine-related health beliefs.

Moderators	Self-reported mental disorder (0 = no, 1 = yes)			Depressive symptoms (PHQ-2; 0 = nonclinical, 1 = clinical)			Anxiety symptoms (GAD-2; 0 = nonclinical, 1 = clinical)		
	β	95% *CI*	*p*	β	95% *CI*	*p*	β	95% *CI*	*p*
Primary outcomes									
Vaccination willingness	< 0.01	−0.08, 0.08	0.990	−0.06	−0.13, 0.02	0.153	−0.04	−0.12, 0.03	0.269
Awareness	−0.01	−0.08, 0.07	0.859	−0.05	−0.13, 0.03	0.201	−0.03	−0.11, 0.05	0.525
Secondary outcomes									
*Threat perceptions*									
Severity	−0.01	−0.09, 0.06	0.728	−0.07	−0.15, < 0.01	0.064	< 0.01	−0.08, 0.08	0.969
Susceptibility	0.09	0.02, 0.17	0.016^*^	0.02	−0.05, 0.10	0.549	< 0.01	−0.08, 0.07	0.926
*5C dimensions*									
Confidence	−0.04	−0.12, 0.03	0.260	−0.03	−0.11, 0.05	0.444	−0.02	−0.10, 0.06	0.625
Complacency	0.02	−0.06, 0.09	0.671	0.04	−0.04, 0.11	0.342	−0.01	−0.09, 0.07	0.872
Constraints	−0.01	−0.09, 0.07	0.850	0.02	−0.05, 0.10	0.554	−0.01	−0.09, 0.07	0.820
Calculation	−0.03	−0.10, 0.05	0.521	< 0.01	−0.07, 0.08	0.925	0.02	−0.06, 0.10	0.610
Collective responsibility	−0.05	−0.13, 0.03	0.186	−0.04	−0.12, 0.04	0.315	0.02	−0.06, 0.10	0.569

All outcomes were measured on a seven-point Likert scale (1–7). Moderator x condition interaction, controlled for age and gender. ^*^p < 0.05.

### Subgroup analyses

Subgroup analyses of 51 (8.0%) unvaccinated participants, including 9 individuals reporting a mental disorder, indicated descriptively more favorable values for several outcomes in the intervention group (e.g., disease-related threat perceptions, confidence). However, no statistically significant effects of the video intervention were observed on any primary or secondary outcomes within this subgroup. Due to the limited sample size of the subgroup, moderation analyses were not performed as intended. Detailed results of the subgroup analyses are provided in Supplementary Table 4.

## Discussion

This study evaluated the effectiveness of a video-based educational intervention aimed at enhancing COVID-19 vaccination willingness and supporting associated health beliefs during the later stages of COVID-19 vaccine distribution in Germany. The intervention emphasized the heightened necessity of vaccination for individuals with mental disorders, aiming to address their increased vulnerability to SARS-CoV-2 infection and severe disease outcomes (8, 9, 40). In line with our hypothesis, our findings indicate small effects in increasing general awareness of the necessity for COVID-19 vaccination for individuals with mental disorders and perceived severity of COVID-19 in the total sample. Additionally, a differential increase in perceived susceptibility was observed among participants reporting a mental health diagnosis. The observed effects are consistent with prior findings on video-based educational interventions to promote COVID-19 vaccination acceptance (32). Notably, the moderating effect of the self-reported presence of any mental disorder on perceived susceptibility highlights the potential of tailored communication. This suggests that individuals may process health risk information differently when specifically addressed. Despite their objectively higher risk, previous studies have documented lower vaccination coverage and reduced perceptions of susceptibility in individuals with mental disorders (14, 16). The present findings suggest that a short educational video highlighting the importance of COVID-19 vaccination effectively targeted this gap by enhancing perceived susceptibility in this group. However, no differential effects were observed for participants experiencing heightened internalizing psychopathology (e.g., depressive or anxiety symptoms). This may indicate that diagnostic status rather than experiencing psychopathological symptom burden was a critical factor for individuals to perceive themselves as directly targeted by the intervention. Future interventions could adopt a broader framing to effectively engage individuals experiencing significant psychological distress, regardless of diagnostic status.

Evidence was also found for a small effect on calculation indicating a promotion of evaluative aspects of vaccine-related decision-making. While calculation is theoretically posited to be negatively associated with vaccine uptake due to its supposed links with heightened risk aversion and susceptibility to misinformation (20), it was negatively associated with intention and confidence in the present study. This finding raises questions about potential negative effects of the video intervention on motivational aspects. In a previous investigation, calculation did not distinguish between individuals who were unvaccinated, partially vaccinated, or had received booster doses (23), thus, playing a relatively minor role in shaping vaccination intention compared to the other antecedents under study (41).

Contrary to our expectations, the intervention did not result in significant changes in vaccination willingness or vaccine-specific beliefs. These findings diverge from the results of a prior video-based educational intervention (32) but align with the absence of effects observed in comparable text-based interventions targeting COVID-19 vaccination intent (42, 43). Several factors may account for these null findings. The psychological antecedents examined in this study may vary in their susceptibility to modification. For example, a study investigating the first 2 years of vaccine distribution in Germany found that disease-related threat perceptions exhibited greater temporal variability compared to vaccine-specific beliefs (e.g., confidence), which remained relatively stable both within and across individuals (16). Furthermore, these stable beliefs may have been particularly resistant to change during the progressed stage of vaccine distribution when the study was conducted. As we suggest a societal deficiency in awareness regarding the heightened risks of COVID-19 faced by individuals with mental disorders, risk evaluations may be more amenable to correction than pre-existing attitudes. Integrating dissonance-based interventions or narratives into educational approaches may be promising to better address vaccine-specific beliefs (44, 45).

Moreover, the sample predominantly exhibited favorable attitudes toward COVID-19 vaccination, with four out of five participants already being fully vaccinated. This imbalance may have introduced a ceiling effect, thereby limiting the potential for the intervention to generate measurable changes. While descriptive data suggested more favorable outcomes for some antecedents within the subgroup of unvaccinated individuals, the study lacked sufficient statistical power to detect significant effects in this critical target population. Importantly, evidence supporting the effectiveness of educational interventions among vaccine-hesitant individuals remains generally weak [e.g., (42)], raising questions about the suitability of indirect educational approaches in addressing entrenched vaccine hesitancy. This outcome is closely tied to the universal prevention strategy employed in the present study. The absence of targeted recruitment strategies and participant screening led to a sample with mental disorder prevalence rates similar to those observed in the general population, but failed to include a representative proportion of vaccine-hesitant individuals. While universal strategies offer broad reach and scalability, these findings highlight limitations of this approach. In contrast, indicated prevention strategies, though more resource-intensive, may be better suited to evaluate targeted communication strategies.

Furthermore, the intervention may not have been adequately processed by participants. The high attrition rate in the intervention condition, likely due to insufficient engagement with the content, suggests that the intervention struggled to sustain viewer attention. Critical information intended to influence vaccination attitudes may have been presented in later segments of the video reducing its impact. Incorporating interactive elements, which have demonstrated effectiveness in enhancing engagement with internet-delivered healthy lifestyle interventions (46), could improve adherence and effectiveness in future interventions. Methodologically, this attrition also resulted in reduced statistical power, thereby limiting the ability to detect small effects with the targeted probability of 80%.

### Limitations

Further limitations should be considered when interpreting the results of this study. First, the use of single-item measures, some of which were partially self-developed, may have limited the reliability and validity of the constructs being assessed. However, while single-item measures may not fully capture complex psychological constructs, they can be more parsimonious and reduce participant burden (47). We argue that the use of single-item measures is appropriate given the relatively short duration of the intervention and the narrow scope of the outcomes, as compared to other psychological constructs (48). Intercorrelations in the current study further support evidence of construct validity of our outcome measures. Second, mental disorders were assessed through self-report and measures of dimensional psychopathology lacked symptoms on the externalizing or thought disorder spectrum, which limits the validity of differentiating our target group from the general population. Third, the study design did not incorporate repeated measurements, resulting in the absence of baseline values for outcomes and the inability to assess long-term effects. This lack of longitudinal data limits our ability to draw conclusions about the persistence of any observed effects over time and to control for pre-intervention differences between groups. Fourth, the study did not include behavioral measures of vaccination, relying instead on self-reported intentions and related psychological antecedents. While intentions are often strong predictors of behavior, they do not always translate into actual vaccine uptake (49). Thus, findings of the present study are limited to cognitive prerequisites of vaccination rather than concrete behavioral changes. Fifth, the absence of an active control condition represents a further limitation. As participants in the control group did not receive a video, it remains unclear whether the observed effects can be attributed to the specific content of the intervention or to non-specific effects of video exposure. Sixth, the final sample size permitted the detection of small effects with a statistical power of 71.4%, which falls below the conventional threshold of at least 80%. Lastly, the generalizability of the findings is constrained by the aforementioned selection bias, which led to an overrepresentation of participants who were already vaccinated and held favorable attitudes toward COVID-19 vaccination. Additionally, sample distributions of gender and educational level of the sample were not representative of the German general population. Generalizability is further limited with respect to other temporal contexts, as data collection was conducted within a relatively narrow time frame. Whereas prior evidence predominantly reflected earlier stages of the pandemic, the present study provides rare insights into the modifiability of vaccine-related health beliefs during a phase characterized by efforts to increase booster vaccination. However, the findings do not permit conclusions regarding post-pandemic settings in which the perceived threat of COVID-19 has substantially diminished.

## Conclusion

The development of effective interventions to promote positive health behavior outcomes is essential for addressing public health crises such as the COVID-19 pandemic. The present video-based educational intervention demonstrated the effectiveness in increasing awareness of the importance of COVID-19 vaccination for individuals with mental disorders and enhancing risk evaluations, particularly within this high-risk group, even during the advanced stages of vaccine distribution. Although the observed effect sizes were modest, they remain practically significant given the intervention's low intensity, cost-effectiveness, and scalability (50). However, significant changes in vaccination willingness and vaccine-specific beliefs were absent. While the intervention was designed to primarily target vaccine-hesitant individuals with mental disorders, the universal prevention approach adopted in the study resulted in a sample that did not adequately represent this high-risk group. As a result, it was not possible to draw definitive conclusions regarding the impact of the intervention on the intended population. These findings suggest that indicated prevention strategies may be better suited to evaluate targeted communication strategies.

## Data Availability

The datasets presented in this study can be found in online repositories. The names of the repository/repositories and accession number(s) can be found below: https://doi.org/10.23668/psycharchives.22187.
