# Supplementary material for: Effectiveness of a video-based intervention for COVID-19 vaccine acceptance among individuals with mental disorders: a randomized online experiment
Source: Front Public Health. 2026 Jun 23;14:1821349. doi: 10.3389/fpubh.2026.1821349 (PMC13337748; doi:10.3389/fpubh.2026.1821349)

frontiers

Supplementary Material

to the article entitled

Effectiveness of a Video-Based Intervention for COVID-19 Vaccine Acceptance among Individuals with Mental Disorders: A Randomized Online Experiment

by Daniel Huth, Severin Hennemann, Michael Witthöft, and Anne-Kathrin Bräscher

Department of Clinical Psychology, Psychotherapy, and Experimental Psychopathology, Johannes Gutenberg University Mainz

1 Content

Supplementary Table 1 Demographic and clinical characteristics between per protocol and excluded participants of the intervention condition (educational video).

Supplementary Table 2 Frequencies of mental disorder diagnostic categories within the study sample.

Supplementary Table 3 Pearson correlations between study variables.

Supplementary Table 4 Observed means and standard deviations for primary and secondary outcomes per condition and between-group comparisons for unvaccinated participants.

Supplementary Figure 1 Participant flow.

Supplementary Table 1 Demographic and clinical characteristics between per protocol and excluded participants of the intervention condition (educational video).

Variables	Per protocol (*n* = 260)	Excluded (*n* = 76)	Test statistics
Age, years, *M*(*SD*)	36.63 (14.35)	34.84 (14.27)	$t(334) = -0.96$, $p = .340$
Gender, *n*(%)			$\chi^2 = 3.48$, $p = .166$[a]
male	203 (78.1)	54 (71.1)	
female	53 (20.4)	22 (28.9)	
other	4 (1.5)	0 (0)	
Education, *n*(%)			$\chi^2(2) = 4.74$, $p = .093$
Secondary school degree or lower	29 (11.2)	9 (11.8)	
Higher education entrance qualification	93 (35.8)	37 (48.7)	
University degree	138 (53.1)	30 (39.5)	
Prior SARS-CoV-2 infection, yes, *n*(%)	90 (34.6)	30 (39.5)	$\chi^2(1) = 0.41$, $p = .521$
COVID-19 vaccine doses, *n*(%)			$\chi^2 = 1.98$, $p = .726$[a]
0	22 (8.5)	7 (9.2)	
1	2 (0.8)	1 (1.3)	
2	24 (9.2)	10 (13.2)	
3	197 (75.8)	52 (68.4)	
4	15 (5.8)	6 (7.9)	
Self-reported mental disorder, yes, *n*(%)	66 (25.4)	18 (23.7)	$\chi^2(1) = 0.02$, $p = .880$
Depressive symptoms			
PHQ-2, sum, *M*(*SD*)	1.79 (1.63)	1.53 (1.49)	$t(334) = -1.26$, $p = .210$
PHQ-2, clinical[b], *n*(%)	70 (26.9)	15 (19.7)	$\chi^2(1) = 1.25$, $p = .264$
Anxiety symptoms			
GAD-2, sum, *M*(*SD*)	1.69 (1.54)	1.82 (1.69)	t(334) = 0.60, $p = .548$
GAD-2, clinical[b], *n*(%)	59 (22.7)	22 (28.9)	$\chi^2(1) = 0.94$, $p = .333$

Note. *n* = 336. [a]Chi-squared test with simulated *p*-value based on 2000 replicates, [b]Clinical values are reached at sum ≥ 3.

Supplementary Table 2 Frequencies of mental disorder diagnostic categories within the study sample.

Diagnostic categories	*n*(%)
Major depressive disorder	94 (14.7)
Anxiety disorder	53 (8.3)
Posttraumatic stress disorder	30 (4.7)
Personality disorder	19 (3.0)
Eating disorder	17 (2.7)
Attention deficit hyperactivity disorder	16 (2.5)
Chronic pain	12 (1.9)
Somatic symptom disorder	12 (1.9)
Other	28 (4.4)

Note. *n* = 639. Frequencies are based on self-reported mental disorders that have been diagnosed by a professional. Participants could select multiple responses.

Supplementary Table 3 Pearson correlations between study variables.

		1	2	3	4	5	6	7	8	9	10	11	12
**1**	Age	-	-0.15**	-0.20***	0.08	<0.01	0.07	-0.04	-0.01	-0.07	-0.09	0.19***	0.04
**2**	Depression, PHQ-2	-0.04	-	0.69***	-0.03	-0.05	0.14**	0.27***	-0.07	0.09	0.05	0.04	-0.04
**3**	Anxiety, GAD-2	-0.07	0.72***	-	-0.01	-0.03	0.09	0.26***	-0.09	0.10	0.08	-0.05	-0.03
**4**	Intention	0.03	-0.14*	-0.06	-	0.82***	0.43***	0.15**	0.75***	-0.75***	-0.21***	-0.17***	0.51***
**5**	Awareness	0.02	-0.11	-0.07	0.84***	-	0.46***	0.18***	0.75***	-0.75***	-0.22***	-0.10	0.55***
**6**	Severity	0.15*	0.07	0.08	0.53***	0.56***	-	0.36***	0.40***	-0.50***	-0.14**	0.01	0.31***
**7**	Susceptibility	0.07	0.24***	0.21***	0.17**	0.18**	0.28***	-	0.19***	-0.17***	-0.05	0.01	0.13**
**8**	Confidence, 5C	-0.02	-0.14*	-0.10	0.80***	0.79***	0.47***	0.14*	-	-0.68***	-0.19***	-0.10*	0.50***
**9**	Complacency, 5C	-0.12	0.12*	0.08	-0.65***	-0.72***	-0.52***	-0.17**	-0.68***	-	0.33***	0.09	-0.64***
**10**	Constraints, 5C	-0.13*	0.11	0.11	-0.23***	-0.22***	-0.17**	0.01	-0.14*	0.34***	-	-0.03	-0.42***
**11**	Calculation, 5C	0.14*	0.08	0.01	-0.17**	-0.14*	-0.03	0.03	-0.17**	0.17**	-0.01	-	-0.08
**12**	Collective responsibility, 5C	0.05	-0.09	-0.09	0.62***	0.60***	0.45***	0.13*	0.55***	-0.62***	-0.42***	-0.09	-

Note. Correlations of the control condition (no video; n = 379) are displayed above the diagonal, correlations of the intervention condition (educational video; n = 260) are displayed below the diagonal. *p < .05, **p < .01, ***p < .001.

Supplementary Table 4 Observed means and standard deviations for primary and secondary outcomes per condition and between-group comparisons for unvaccinated participants.

Outcomes	Video (n = 22)	Control (n = 29)	Effect size d [95% CI]
**Primary outcomes**			
Vaccination willingness	1.27 (0.63)	1.28 (0.92)	0.01 [-0.56, 0.58]
Awareness	2.45 (1.53)	2.21 (1.61)	0.14 [-0.41, 0.70]
**Secondary outcomes**			
Threat perceptions			
Severity	3.41 (1.53)	2.90 (1.21)	0.37 [-0.21, 0.95]
Susceptibility	3.09 (1.87)	2.66 (1.49)	0.24 [-0.31, 0.80]
5C dimensions			
Confidence	1.82 (1.47)	1.41 (0.87)	0.34 [-0.23, 0.92]
Complacency	4.00 (1.57)	4.66 (1.67)	-0.43 [-0.95, 0.10]
Constraints	1.55 (1.18)	1.31 (0.81)	0.23 [-0.33, 0.80]
Calculation	6.14 (1.49)	6.28 (1.58)	-0.09 [-0.67, 0.49]
Collective responsibility	4.45 (1.84)	4.76 (1.75)	-0.15 [-0.71, 0.40]

Note. All outcomes were measured on a seven-point Likert scale (1-7). Main effects of condition were estimated controlling for age and gender.

Supplementary Figure 1 Participant flow.

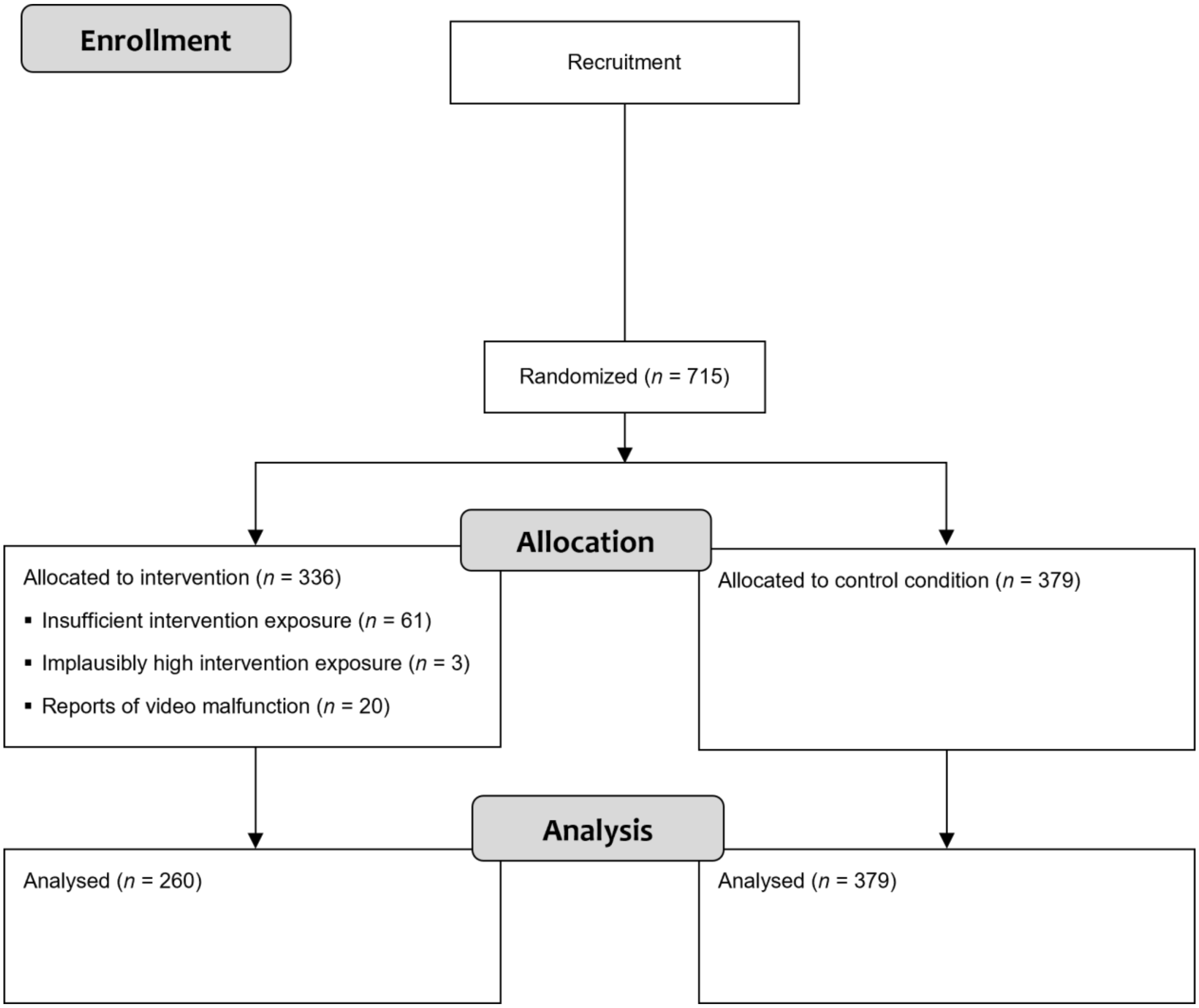

Supplement: Supplementary file 1 [file Supplementary_file_1.pdf]
